# Ministère de l'Intérieur

**Published:** 1804-10-01

**Authors:** John Walker

**Affiliations:** Salisbury Square


### *To the Editors of the Medical and Physical Journal.*

GENTLEMEN,

I Request you to communicate, for the information of your every-where spread Readers, the reception which Paris, as well as Edinburgh and Dublin, has given to the pamphlet of William Goldson. Your insertion of the following official paper will also tend to show the pains which have been taken to give it gratuitous diffusion.

*Salisbury Square; 20, ix, 1804.* JOHN WALKER.

---

### Ministère de l'Intérieur.

*Paris, le 8 Messidor, An. 12.*

LE SECRETAIRE DU COMITÉ CENTRAL DE VACCINE, à Mons. NOWELL, Physician, No. 100, Great St. Martin's Lane, Charing Cross, London.

Monsieur,

La Société vient de recevoir par les soins de M. Blagden un petit ouvrage, intitulé, "Cases of Small-pox subsequent to Vaccination, with Facts and Observations: read read before the Medical Society at Portsmouth, March 29, 1804. Addressed to the Directors of the Vaccine Institution. By William Goldson, Member of the Royal College of Surgeons, in London. Portsea, 1804.

Plusieurs faites contenus dans cette brochure méritent toute notre attention, & nous n'osons y ajouter foi qu'après avoir appris de vous quelle peut être la cause des evenemens qui y sont rapportés. Nous ignorons quelle confiance on doit avoir dans les assertions de M. Goldson; et jusqu'a ce que vous nous ayez instruit des moindres details, il est bon que nous nous tenions sur la defensive. Ces faits, s'ils existent, nous paraissent tellement extraordinaire, si peu conformes à tous ceux observés depuis longtemps dans tout le monde savant, que nous n'hesitons pas à croire qu'il y a eu quelqu' erreur commise dans la vaccination, ou qu'on a pu se tromper sur l'inoculation de la petite verole.

Quoiqu'il en soit, Monsieur, la nouvelle Société ajoute beaucoup d'importance à recevoir de vous tous les détails qui pourront l'éclairer, et elle ne doute pas que la plus grande impartialité ne vous guide dans la reponse que je suis chargé de vous supplier de vouloir bien lui faire.

Vous ne pouvez douter, Monsieur, de l'empressement avec lequel je saisis cette occasion de vous assurer de ma haute consideration.

TURRON.

